# A partial encryption algorithm for medical images based on quick response code and reversible data hiding technology

**DOI:** 10.1186/s12911-020-01328-2

**Published:** 2020-12-15

**Authors:** Jian Li, Zelin Zhang, Shengyu Li, Ryan Benton, Yulong Huang, Mohan Vamsi Kasukurthi, Dongqi Li, Jingwei Lin, Glen M. Borchert, Shaobo Tan, Gang Li, Bin Ma, Meihong Yang, Jingshan Huang

**Affiliations:** 1 Qilu University of Technology (Shandong Academy of Science), Shandong Provincial Key Laboratory of Computer Networks Jinan, Jinan, China; 2 School of Computing, University of South Alabama, Mobile, AL 36688 USA; 3 College of Allied Health Professions, University of South Alabama, Mobile, AL 36608 USA; 4 Ocean School, Fuzhou University, Fuzhou, China; 5 College of Medicine, University of South Alabama, Mobile, AL 36688 USA

**Keywords:** Image segmentation, Key region, QR code, Reversible data hiding, Selective encryption, Texture complexity

## Abstract

**Background:**

Medical image data, like most patient information, have a strong requirement for privacy and confidentiality. This makes transmitting medical image data, within an open network, problematic, due to the aforementioned issues, along with the dangers of data/information leakage. Possible solutions in the past have included the utilization of information-hiding and image-encryption technologies; however, these methods can cause difficulties when attempting to recover the original images.

**Methods:**

In this work, we developed an algorithm for protecting medical image key regions. Coefficient of variation is first employed to identify key regions, a.k.a. image lesion areas; then additional areas are processed as blocks and texture complexity is analyzed. Next, our novel reversible data-hiding algorithm embeds lesion area contents into a high-texture area, after which an Arnold transformation is utilized to protect the original lesion information. After this, we use image basic information ciphertext and decryption parameters to generate a quick response (QR) code used in place of original key regions.

**Results:**

The approach presented here allows for the storage (and sending) of medical image data within open network environments, while ensuring only authorized personnel are able to recover sensitive patient information (both image and meta-data) without information loss.

**Discussion:**

Peak signal to noise ratio and the Structural Similarity Index measures show that the algorithm presented in this work can encrypt and restore original images without information loss. Moreover, by adjusting the threshold and the Mean Squared Error, we can control the overall quality of the image: the higher the threshold, the better the quality and vice versa. This allows the encryptor to control the amount of degradation as, at appropriate amounts, degradation aids in the protection of the image.

**Conclusions:**

As shown in the experimental results, the proposed method allows for (a) the safe transmission and storage of medical image data, (b) the full recovery (no information loss) of sensitive regions within the medical image following encryption, and (c) meta-data about the patient and image to be stored within and recovered from the public image.

## Background

Research in medical imaging has progressed at remarkable rates, partially as a result in the increasing investments and enhancements in multimedia technologies. As a result, medical images are an indispensable and effective secondary source of information when medical personnel need to diagnosis a patient [1]. Unfortunately, the quickest (and generally efficient) means of sharing medical images typically is via open networks, such as file share and/or emails. These types of transmissions expose the images to activities such as content tampering, illegal copying, and copyright loss [2]. As a result, research in medical image security has grown, with some foci emphasis in the areas of image encryption and information hiding [3].

Information hiding technology embeds information that needs to be protected into a carrier. Usually, after extracting the secret information, the carrier inevitably exhibits a certain degree of distortion. However, special carriers such as medical images have extremely high requirements for image integrity. To solve this problem, reversible data hiding (RDH) technology, which was proposed by Barton [4] in 1997, is typically introduced to protect highly sensitive images. To hide data, RDH modifies the carrier data such that it is possible to extract all of the hidden information while enduring there is no corruption to the carrier data [5–7]. The carrier data can be another image, a sound file, or any other digital artifact. A number of different methods and algorithms for reversible data hiding have been proposed [8–10]. At present, reversible data hiding technology mainly includes reversible data hiding technology based on lossless compression, reversible data hiding technology based on Difference Expansion (DE), and reversible data hiding technology based on Histogram Shifting (HS). Celik et al. [11] presented a scheme, which has a low computational cost that utilizes generalized least significant bit embedding, image quantization and residuals to ensure reversible data hiding. In 2003, Tian [12] suggested a RDH method that permits a multi-layered embedding of the secret data into the cover layer. This is achieved, in part, by using difference expansion to allow multiple bit adjustments, creating a location map to guide the decoding algorithm for each layer (based on work by Ni et al. [13]), and, in addition to embedding the secret information, but also carefully embedding the location map and information needed to ensure correct reconstruction of the cover data (image). Kumar [14] et al. proposed an improved HS reversible medical image watermarking algorithm to improve the hidden capacity. It divides the image into smaller blocks for data embedding based on HS techniques. Yang et al. [15] prioritizes data embedding into texture areas by HS and contrast enhancement methods, enhancing the contrast of texture areas and improving subjective perceived image quality. Wu et al. [16] uses a reversible image hiding scheme for HS to protect patient information after which two parameters of the linear predictor with weights and thresholds are applied to improve medical image quality and embedding rate. Huang et al. [17] proposed a HS method for image reversible data hiding testing on high bit depth medical images. Among image local block pixels, we exploit the high correlation for smooth surface of anatomical structure in medical images. It is very easy to adjust capacity, peak signal to noise ratio (PSNR) by block size, partition level, and number of embedding bits. A goal of many of the above methods was to ensure image copyright protection and minimize the distortion of the visual quality of the embedded image.

Due to the reversible nature of the reversible data hiding algorithm, the use of reversible data hiding for medical image protection has important practical significance. As above, most protection algorithms that deal with medical images are principally concerned with image copyright protection and embedded image visual quality enhancement. The security of the image content itself, especially when it is also the cover source, is often not a key concern. If the image content is changed, the information hiding methods will become damaged, indicating the compromise of the cover image (and the secret data). Hence, to protect alterations of the image content (and any accidental disclosures it may bring), most medical images are encrypted when transmitted and/or stored.

The purpose of image encryption technology is to turn a given image into a disorganized image according to a certain mathematical calculation. Protection of image content in this manner is strong, and encrypted images are highly secure. Norcen et al. [18] recently examined the storage and transmission of medical image data. In short, two AES-based selective encryption and partial encryption techniques were proposed: RST encrypts the bit-plane subset of normal image data, and encrypts the portion of the JPEG2000 bitstream. When image is encrypted using selective bit-plane, up to 50% of the data need encryption, while in the case of JPEG2000 bitstream encryption, protection of 20% of the data has produced satisfactory results. This huge difference is due to the fact that important visual features are concentrated at the beginning of the embedded JPEG2000 bitstream, so that effective protection is achieved, whereas visual features are largely dispersed in the bit plane. Brahimi et al. [19] also presented a selective encryption image scheme primarily based on JPEG2000. In this case, the method only encrypts regions that contain the sensitive information, which often is only a subset of the image. Experiments indicated that the size of the image is not modified and that using both permutation and encryption together to protect the sensitive regions lead to the lower PSNR. PSNR is typically used as measure the image quality, where lower PSNR indicates more distortion/noise. For this application, trying to hide what is in the sensitive areas, a low PSNR means more distortion, which makes it harder to visualize what is present. In a somewhat different approach, Abdel-Nabi et al. [20] divides the image into two segments; one segment has a watermark embedded first and then is encrypted, while the second segment is encrypted and then has a watermark applied. The second watermark can be used to allow verification that the encrypted file has not been modified (tampered); the first watermark allows the end user to know that the image itself hasn’t been modified during encryption/decryption. A drawback, of course, is the watermarks lower the amount of additional data that can be embedded into the image. It should be noted that, for all the previously mentioned approaches, the ability to embed patient information into the images is limited to the available payload of the images, which is typically partially used to encode data to ensure lossless recovery. Moreover, retrieval of the images, within an open network, can be problematic, unless there is additional non-encrypted information that can be accessed (which could compromise some of the effort of protection) and a mechanism by which only authorized medical personnel can decrypt information.

To address some of these issues, we propose a medical image protection algorithm that (a) encrypts sensitive information, (b) enables patient information embedding, and (c) produces a modified image that is the same size as the original. In addition, the proposed approach offers protection of both medical image copyrights and patient information. The proposed approach is able to achieve this, in part, through the use and exploitation of a quick response (QR) code [21].

**Fig. 1:**
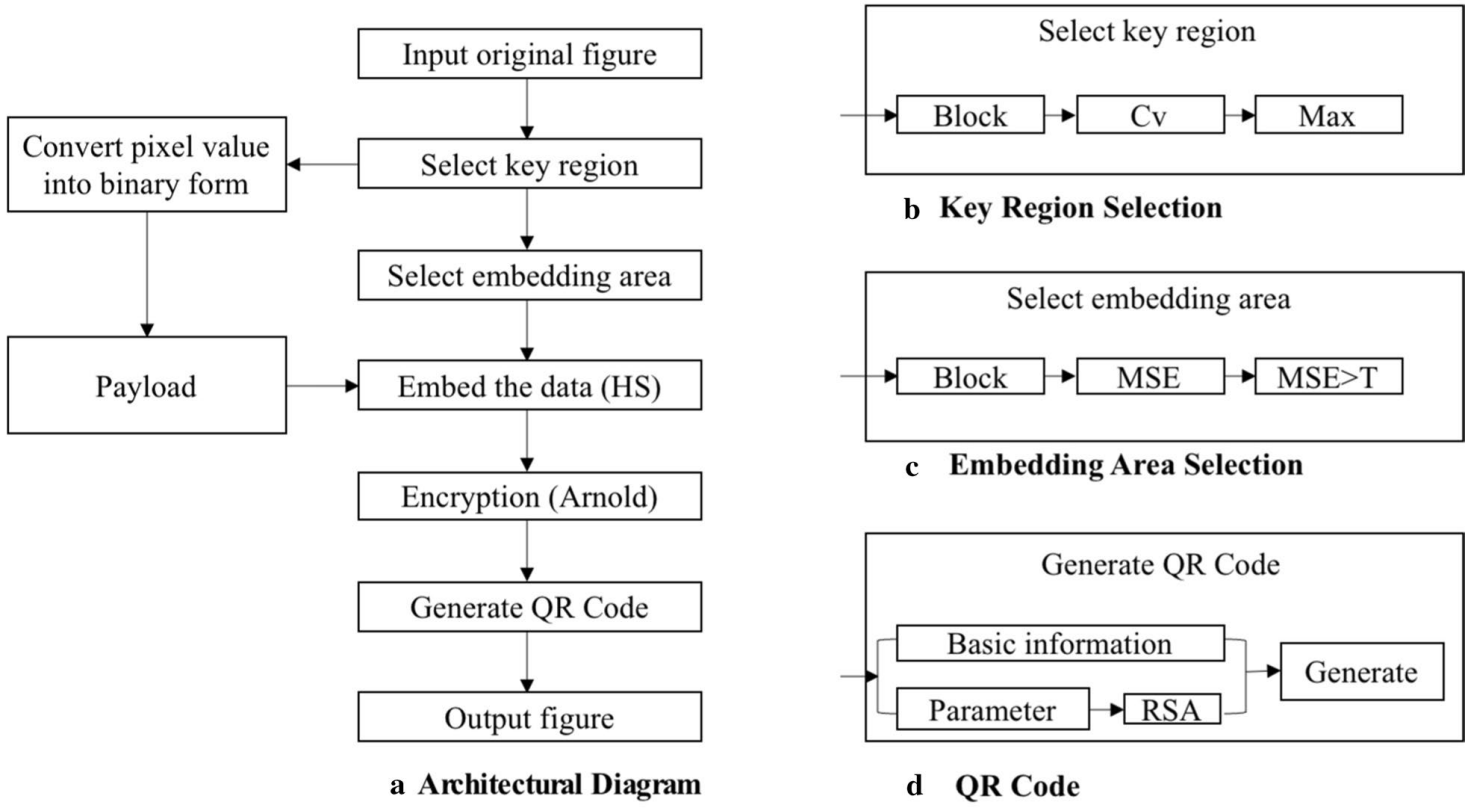
General structure diagram

**Fig. 2:**
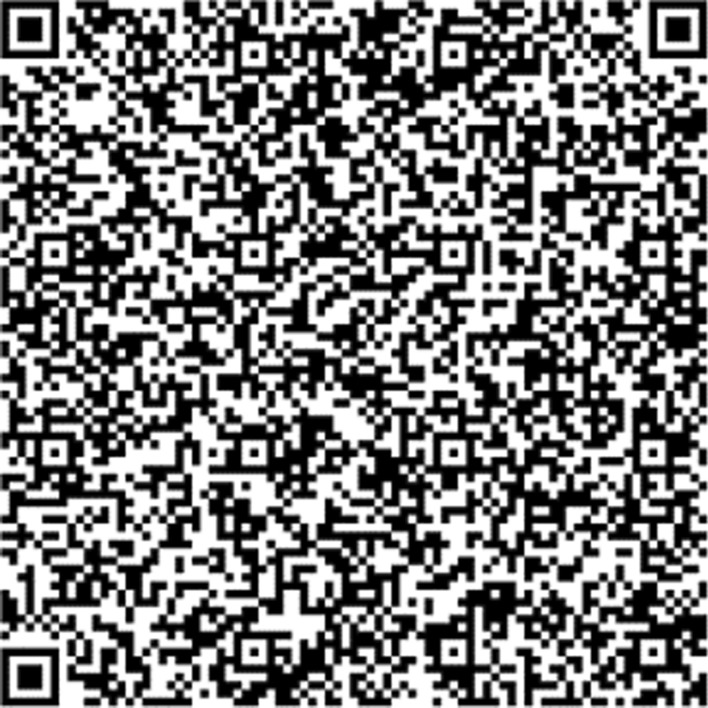
QR code. This is an example of the QR code generated by the basic image information of a computerized tomography

**Fig. 3:**
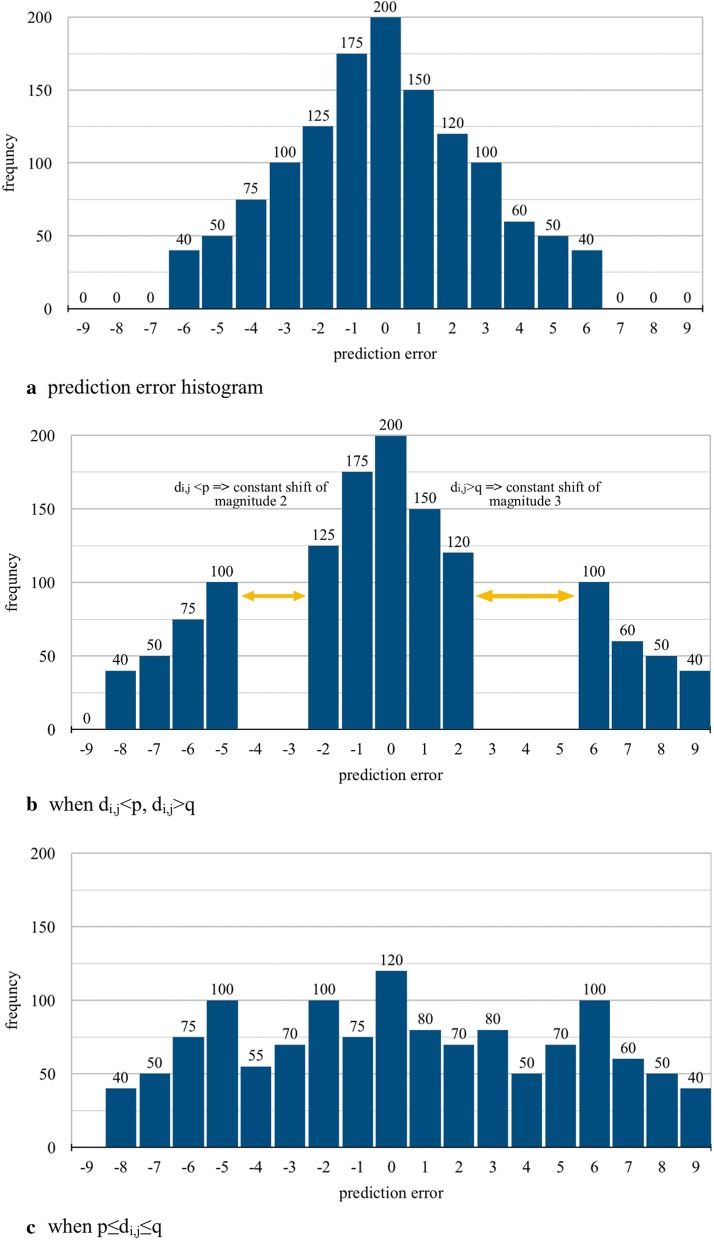
Prediction error expansion (prediction error = [− 2, 2])

This manuscript is an extension of our previously published work [22]. Compared with the original paper, most sections have been significantly expanded in this version. Major extensions (not including minor modifications) are summarized as follows. (1) Background: expanded the introduction of image encryption technology along with five references. (2) Methods: expanded the algorithm flow, added three figures (Figs. 1, 2, and 3) and four references; added details on using Mean Squared Error (MSE) to sort image blocks; in addition, a Prediction Error Expansion (PEE) and QR code section have been added; a new detailed example of QR code generation; and an explanation of the role QR codes play in this algorithm. (4) Results and Discussion: Fig. 6 was added to better illustrate an encrypted versus decrypted image; Fig. 7 was added to demonstrate unauthorized use effects; new experiments showing the effects of using different thresholds and three accompanying figures (Figs. 9, 10, and 11) were also added; Table 3 was added to show the experimental consequences of choosing a different mean square error range; and finally, Table 4 was added to provide a comparison of our algorithm with other existing methods. Our most significant work is to combine QR code technology with encryption technology to provide ciphertext image retrieval, and use reversible data hiding technology to strengthen image security so that only authorized customers are able to obtain an encryption key and extract information from the encrypted image.

The rest of this paper is structured as follows. In the “Methods” section, we detail our medical image protection algorithm step by step. In the “Results and discussion” section, we report the experimental results from this study and detailed discussion. Finally, we conclude with future work.

## Methods

Figure 1 introduces the architecture of our method. It is composed of four parts. Figure 1a is a flow chart of general architecture. Figure 1b is the sub-diagram for selecting the key region scheme. Figure 1c shows the steps for selecting the embedded region scheme, and Fig. 1d describes the process of generating the QR code. First, coefficient of variation is employed to identify key regions, a.k.a. image lesion areas; then additional areas are processed as blocks and the texture complexity is analyzed. After that, our novel reversible data-hiding algorithm embeds lesion area contents into a high-texture area, and an Arnold transformation is utilized to protect original lesion information. Finally, we use basic image information and decryption parameters to generate a QR code and replace original key regions.

### Information encryption

Figure 1a describes the overall encryption process of the algorithm. The steps are as follows:

- *Step 1*: Select the key region. The image is divided into 160 × 160 minimum units then the key image block is selected. To achieve this, the *C*_*v*_ of each image block is calculated and the block with the largest *C*_*v*_ value is selected as the key region. Convert each key region pixel value into binary form then use as the secret information to be embedded.
- *Step 2*: Select the embedded area according to the selection scheme. We employ a trial and error method to find a suitable threshold trying all potential thresholds at each increment of five from five up to 50 as detailed in Figs. 9, 10, and 11.
- *Step 3*: The binary stream transformed by the key region image block is embedded into the selected embedded region using an improvement of HS: prediction error expansion.
- *Step 4*: Arnold transformation on the embedded area is carried out, with an embedded area being divided into individual 8 × 8 image blocks.
- *Step 5*: Image basic information and ciphertext section obtained with RSA encryption, Arnold transformation-related parameters are used to generate a QR code of the same size as a key region. The QR code replaces a key region to generate the encrypted image.

### Key region selection scheme

This section is a detailed introduction to Step 1 of Information Embedding section. Figure 1b illustrates the process of selecting the key region scheme. In order to effectively protect the contents of medical images, key regions are extracted and embedded into the original images as secret information. The key region, which is typically a disease precursor region, is one that provides diagnostic information such as important pathological features, which provide information needed diagnosis and treatment. These regions are often rich in texture, which implies high information content. To measure the richness of the region, the coefficient of variation (*C*_*v*_) can be calculated, which is the degree of dispersion normalized measure. The greater the coefficient value, the larger amount of information the region contains. The smaller it is, the more uniform the region; in other words, less variation exists in the pixel values. *C*_*v*_ is determined according to:

$$C_{v} = \frac{\sigma }{\mu }$$

*σ* means the standard deviation, and *μ* means the overall average.

Given that the key region is assumed to be rich in texture, an assumption is made that the key region will be the region with the highest coefficient of variation. Using this assumption, the process of identifying the key region can be automated. To do this, the original medical image is split into a set of non-overlapping blocks, where each block is a specified size. Next, the *C*_*v*_ value of individual blocks is calculated. The block with the greatest *C*_*v*_ value is then selected to be the key region. The standard deviation *σ* is calculated using:

$$\sigma = \sqrt {\frac{{1}}{N}\sum\limits_{{{\text{i}} = 1}}^{N} {\left( {x_{i} - \mu } \right)^{2} } }$$

where *N* represents the total number of pixels of a pixel block, *x*_*i*_ is the pixel value, and *μ* represents the overall average.

### Select texture area as embedding area

This section is a detailed introduction to Step 2 of Information Embedding section. Figure 1c illustrates the process of selecting the embedded area scheme. MSE is used to assess degrees of image change; in other words, it can be used as an indication of how much texture is present around a given location. The MSE of each image block is calculated according the following equation:

$$MSE = \frac{1}{mn}\sum\limits_{i = 0}^{m - 1} {\sum\limits_{j = 0}^{n - 1} {\left[ {I(i,j) - I_{{{\text{ave}}}} (i,j)} \right]^{2} } }$$

where *n* and *m* respectively represent the columns and rows of the image block, *I* represents an original image, and *I*_*ave*_ represents image block pixel mean value. MSE is sorted in ascending order according to the corresponding image block position. A threshold value *T* is set, which is used to determine if the block is suitable for embedding information. When the image block $MSE > T$, the pixel values of the image block changes obviously; as a result, this is labeled as a texture area. When the image block $MSE \le T$, the pixel value of the image block changes little and is set as a smooth area. For medical images, the smoother texture region contains more information. Embedding secret information into the texture region can improve the security of information.

For an image, there is not only a huge amount of data, but also the correlation between adjacent pixels. Arnold transformation is carried out on the same digital image through repeated iterations, that is, when the iteration number reaches a certain value the original image is restored. This value depends on the size of the image, and the overall reversible characteristic of the algorithm is realized by combining this characteristic with reversible data hiding.

Arnold transformation for embedded areas can scramble pixels in image blocks to different rows and columns, which not only weakens the strong correlation between adjacent pixels, but also makes the mapping relation of different image pixel positions different. In order not to conflict with key regions, 8 × 8 blocks are typically selected as the block scheme of Arnold transformation. Here, the MSE value of an image block is calculated, and all sub-blocks with *MSE* > *0* are scrambled to ensure full encryption of image content outside the background area. The scrambling times and relevant parameters are encrypted and stored in the QR code. While ensuring the security of the image content, the general outline of the image is kept, thus preventing the image from being maliciously attacked and tampered with and avoiding unnecessary network attacks.

### Prediction error expansion

Importantly, the main steps associated with predicting error expansion are illustrated in the 'Embed the data' Step depicted in Fig. 1a, and this section presents a detailed introduction to the components of Step 3 of the Information Embedding section. Firstly of note, Ni et al. [13] previously proposed HS technology to implement the embedding of secret information. In this algorithm, histograms are generated by pixel and embedded by shift. Following this, Sachnev et al. [23] proposed an improved HS reversible algorithm to improve embedding capacity called PEE which changed the pixel value in HS to the prediction error greatly improving embedding capacity. Key PEE steps include:

- *Step 1* Generating the target pixel prediction by using four adjacent pixel points. *v*_*i,j-1*_, *v*_*i,j*+*1*_, *v*_*i-1,j*_, and *v*_*i*+*1,j*_, taken from around each pixel. Next, prediction error *d*_*i,j*_ is taken as the difference between original pixel value *v*_*i,j*_ and prediction value *u*_*i,j*_. The formula for this is:
  $$\left\{ {\begin{array}{*{20}l} {u_{{i,j}} = \frac{{v_{{i,j - 1}} + v_{{i,j + 1}} + v_{{i - 1,j}} + v_{{i + 1,j}} }}{4}} \hfill \\ {d_{{i,j}} = \left\lfloor {u_{{i,j}} - v_{{i,j}} } \right\rfloor } \hfill \\ \end{array} } \right.$$
- *Step 2* Generating a prediction error histogram for each image block then calling pixel points with a prediction error of [− 2, 2] for embedding points. Next, translation resolves embedded space and embedded secret information [24, 25], the formula of this translation is as follows:
  $$D_{i,j} = \left\{ {\begin{array}{*{20}l} {2d_{i,j} + b,d_{i,j} \in [p,q]} \\ {d_{i,j} + q + 1,d_{i,j} > q} \\ {d_{i,j} + p,d_{i,j} < p} \\ \end{array} } \right.$$
  where [*p*, *q*] represents the error selection range and the secret information is represented by *b*.
- *Step 3* The prediction error [24, 25] histogram is generated. Excepting that the value within the selected error range remains unchanged, the other values are shifted to the left and right sides to leave a certain space then the error within the selected error range is moved according to the embedded information. The process is shown in Fig. 3 using the prediction error of [− 2, 2]. The prediction error *d*_*i,j*_ is calculated according to the equation above. When *d*_*i,j*_ > *q*, because the prediction error is [2], *p* equals to − 2 and *q* equals to 2. So, *D*_*i,j*_ = *d*_*i,j*_ + *q* + 1 = *d*_*i,j*_ + 2 + 1 = *d*_*i,j*_ + 3, representing "constant shift of magnitude 3" in Fig. 3b. When *d*_*i,j*_ < p, p equals to − 2. So, *D*_*i,j*_ = *d*_*i,j*_ + p = *d*_*i,j*_ − 2, representing "constant shift of magnitude 2" in Fig. 3b. When *d*_*i,j*_ ∈ [*p*, *q*], *D*_*i,j*_ = 2*d*_*i,j*_ + *b*, *b* is the binary value converted from pixel value of the key area. One example of this situation is represented in Fig. 3c. Note that Fig. 3 shows an example of frequency equal to [0, 200] whereas in a real experiment frequencies vary according to image.

### Arnold transformation

This section presents a detailed introduction to the components of step 4 of the Information Embedding section depicted in Fig. 1a. Scrambling is a commonly used image encryption technique. It is a kind of way to perturb the pixels within an image. In other words, the image is scanned and the components and/or pixel values (gray or color) are moved resulting in the reduction of nearby pixel correlations. This creates “noise” within the image, and, without knowing the rules, extracting meaning from the image becomes nearly impossible.

Scrambling techniques are generally periodic and do not change image size (only scrambles the pixel order of the image). Moreover, scrambling technology is reversible, and a scrambled image can be recovered completely lossless. In scrambling technology, Arnold transformation has been widely studied and the theoretical system is relatively complete [26–28]. Therefore, we have elected to adopt Arnold transformation in our method to encrypt images.

In brief, the image pixel transformation theory Arnold transformation [29] is the *p* process of first cutting, then splicing, and then rearranging pixels into a resultant square digital image matrix. At the end of the process, a new matrix is obtained, resulting in a scrambled image. *N* is the order (the size) of the digital image matrix, and *I*_*xy*_ represents the gray value of the pixel point with coordinates (*x*, *y*). The transformation is described by the following formula:

$$I_{xy} = \left[ {\begin{array}{*{20}l} {\begin{array}{*{20}l} {I_{11} } \\ {I_{21} } \\ \vdots \\ {I_{N1} } \\ \end{array} } & {\begin{array}{*{20}l} {I_{12} } \\ {I_{22} } \\ \vdots \\ {I_{N2} } \\ \end{array} } & {\begin{array}{*{20}l} \cdots \\ \cdots \\ \ddots \\ \ldots \\ \end{array} } & {\begin{array}{*{20}l} {I_{1N} } \\ {I_{2N} } \\ \vdots \\ {I_{NN} } \\ \end{array} } \\ \end{array} } \right]$$

$$\left( {\begin{array}{*{20}l} {x^{\prime}} \\ {y^{\prime}} \\ \end{array} } \right) = \left[ {\begin{array}{*{20}l} {\begin{array}{*{20}l} 1 & 1 \\ \end{array} } \\ {\begin{array}{*{20}l} 1 & 2 \\ \end{array} } \\ \end{array} } \right]\left[ {\begin{array}{*{20}l} x \\ y \\ \end{array} } \right]\left( {\bmod N} \right)\begin{array}{*{20}l} {} & {x,y \in \left\{ {0,1 \ldots N - 1} \right\}} \\ \end{array}$$

where (*x*, *y*) is the coordinate of the pixel in the original image, (*x*′, *y*′) is the coordinate of the pixel in the new image after transformation. It is generally assumed that image is a square image, and the mode is taken to ensure that the position of the scrambled pixel points does not exceed the subscript limit of the image matrix. When performing Arnold transformation on a digital image, the pixel coordinates in the image are shifted and transformed according to the above two formulas, which is equivalent to one-to-one transposition of their coordinate positions. The distribution curve of the whole image gray value is further disturbed by the change of the corresponding position of the pixel coordinates, thus achieving the encryption purpose, although the process, to ensure good encryption, is often repeated a number of times.

### QR code

This section presents a detailed introduction to the basic principles of QR Codes. In 1994, QR code [21] was developed by a Japanese company for tracking automobile parts then later applied to various fields. Compared with other QR bar codes, QR codes have become the most popular bar codes due to their large storage capacity, fast identification speed, strong anti-fouling ability, encryption, and anti-counterfeiting value [30]. The steps for generating QR codes are as follows:

1. *Data analysis* Analyze the input data stream to determine the type of characters to be encoded. QR codes contain multiple encoding modes to encode the input data and generate QR code symbols. When necessary, mode switching can be carried out to more efficiently convert data into binary bit streams.
2. *Data coding* For the chosen mode, data characters are converted into bit streams according to corresponding rules. The generated bit stream is divided into a plurality of code words according to the principle of one for every eight bits. Padding characters are added when necessary to ensure the number of words in the data code are filled according to the version requirement. When mode conversion is required, a mode indicator is added before the new mode starts to perform mode conversion, and a terminator is added after the data sequence.
3. *Error correction coding* According to the code word sequence to be corrected, the code word sequence is first divided into blocks. Next, the corresponding error correction code words are generated in similar units of blocks. These error blocks are written into corresponding error correction code positions behind the data code word sequence.
4. *Construct final information* Data and error correction code words are placed in each block, and the remaining bits are added if necessary.
5. *Arranging modules in a matrix* The image finding pattern (position detection pattern), separator, positioning pattern and correction pattern are put into the matrix together.
6. *Mask* The bitmap of the coded region of the symbol is masked sequentially with eight mask modes, and the eight obtained results are evaluated then the best one selected.
7. *Generate format and version information* Generate format information and version information symbols. Format information is a functional graph containing the level of error correction used by symbols and the mask graph information used for coding areas. The rest of the domain is decoded. Version information is used to represent the series of symbol specifications and to indicate the level of error correction to which the symbol is applied.

### Patient QR code replacement program

This section presents a detailed introduction to the components of step 5 of the Information Embedding section depicted in Fig. 1d which shows the steps of generating the QR Code. Our algorithm generates QR codes from (a) the basic information about a medical image and (b) ciphertext. Medical image basic information includes information from the hospital, department, and doctor, patient number, contact telephone number, image capturing time, and the type of medical image. Ciphertext is generated by applying an RSA encryption algorithm to Arnold transformation parameters; in other words, the ciphertext contains the information needed to undo the Arnold transformation. For instance, assume we have a computerized tomography (CT) scan of a brain. The basic information of the image would be:

Hospital: union hospital.

- Department: internal medicine
- Doctor number: 526
- Patient number: 10,256
- Shooting time: 9/6
- Contact number: 5628***
- Image type: brain CT

The plain text submitted to the Arnold transformation would be ‘8 × 8 4’. This indicates that the encryption area is divided into 8 × 8 image blocks, and the loop is restored four times. The resulting ciphertext is: J9BsIjqSMXvzhlyM9e4Cpo8f04CdX4wMSPQ1pmfblZesqaM/iQQUtLGCdRssNxFvd72v6hgd4dgYRJ6zvxWekw== 

After generating the QR code in Fig. 2 by using the above information, a QR code is positioned into the medical image key region as a 160 × 160 grey image block comprised of 25,600 pixels, which was experimentally found to be a reasonable size. Smaller key area sizes, such as 64 × 64, were tried; however, the smaller sizes made it more difficult to accurately find the lesion area. Conversely, using larger areas, such as 200 × 200, had the impact of reducing the actual embedding capacity to levels insufficient for the required embedding. More information about the images and experiments can be seen in the “Results and discussion” section.

By scanning the QR code, the basic information of the image can be obtained intuitively, which facilitates quick retrieval of the ciphertext image, simplifies patient information, and protects patient privacy. After checking the relevant information, the authorized party can obtain the relevant plaintext information through the key, decrypt the encrypted information, and then restore the original image. The unauthorized party does not have the key, cannot get the decrypted image, and forcibly recovers only a disorganized image. If the image cannot be recovered, it can be proved that the image is illegally destroyed to protect the copyright information.

### Information decryption

Clearly, after an image has been encrypted by the steps above, there must also be a means of decrypting it. As such, the following section details our overall decryption methodology and recovery process. Importantly, after decryption, we find that our resultant decrypted images and their corresponding original images are exactly the same allowing medical personnel to obtain unaltered original figures after been authorized.

- *Step 1* Scan an image QR code to obtain image basic information and ciphertext then decrypt by using a key obtained by authorization to acquire plaintext.
- *Step 2* Plain text decryption (Arnold transformation related parameters) in order to acquire decrypted images.
- *Step 3* Calculate the MSE of each image block, sort from smallest to largest, and then select smooth areas and texture areas according to threshold *T*. When its MSE value is greater than *T*, the image block will be added into a texture region then serves as an embedding region. Among these, a QR code region represents the key region rather than an embeddable region.
- *Step 4* Perform a diamond prediction on each image block embedded region based on an embedding sequence, predict the even layer then odd layer, and then extract secret information through using the equation:
  $$b = D_{i,j} \bmod { 2}$$
- *Step 5* Generate a prediction error histogram for each image block and then translate and recover an image through this equation:
  $$d_{i,j} = \left\{ {\begin{array}{*{20}l} {D_{i,j} /2,d_{i,j} \in [2p,2q + 1]} \\ {D_{i,j} - q - 1,d_{i,j} > 2q + 1} \\ {D_{i,j} - p,d_{i,j} < 2p} \\ \end{array} } \right.$$
- *Step 6* Extracted secret information can next be recombined into data, and the one-dimensional array recombined to 160 × 160 image blocks through employing consistent QR code sizes. Finally, image blocks are merged with the key region in order to reconstitute the original image.

## Results and discussion

The proposed methodology was implemented in MATLAB; specifically, the version used was MATLAB 2016a. We conducted several different experiments to evaluate the system, which were executed upon a workstation with an Intel(R) Core (TM) i7-6700 CPU processor. To begin, we performed two quick experiments as a sanity check of the approach.

### Dataset

We experimented with data from the cancer imaging archive (TCIA) [27] medical imaging database. TCIA is an open-access database of medical images for cancer research. Patients are generally associated with a common disease (such as lung cancer), image morphology (MRI, CT, etc.), or research focus. As all images are larger than 512 × 512, each was preprocessed to the size of 512 × 512.

We selected 200 images from TCIA manually using the following selection schema: (a) images had to depict commonly CT imaged organs such as brain, lung, etc., (b) image texture was required to be fairly complex. For example, if there are only a few points or lines in a figure, it was excluded, and (c) the image of an organ had to occupy the majority of the space of the figure. Importantly, we find our method accurately blocks 70% of the information area found in images selected by this schema. In addition to this, we also decided to enable key region semi-automatic calibration where, a clinician is able to add or adjust a key area to be embedded.

**Fig. 4:**
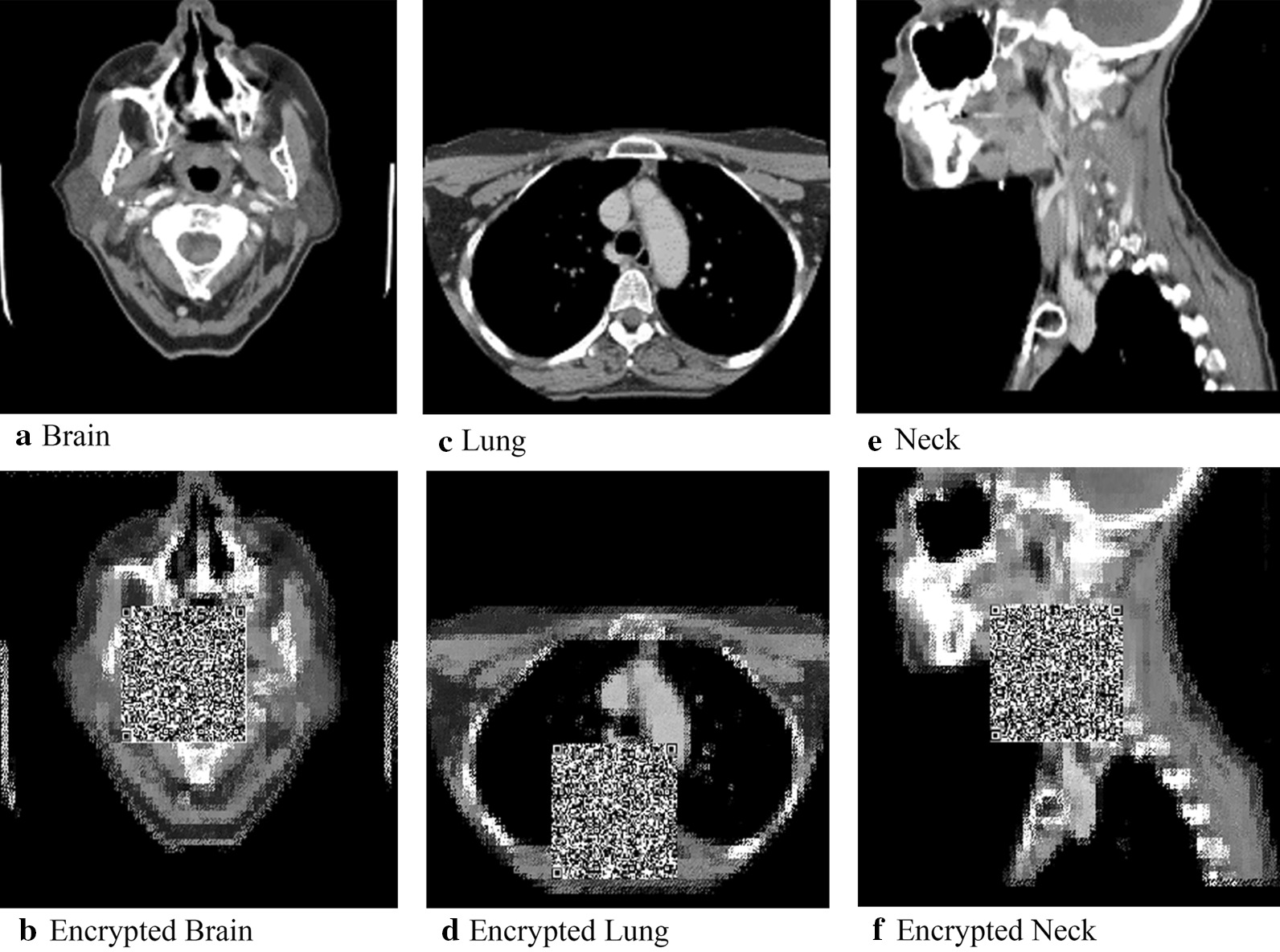
Examples of original and encrypted images

The original images and the resulting encrypted versions are displayed in Fig. 4. As shown in Fig. 4, it can be discerned that, for each medical image, (a) texture areas surrounding the key regions are encrypted, (b) the QR codes cover the key regions, and (c) the pixel positions of the surrounding texture areas are changed.

### Embedded image extraction

**Fig. 5:**
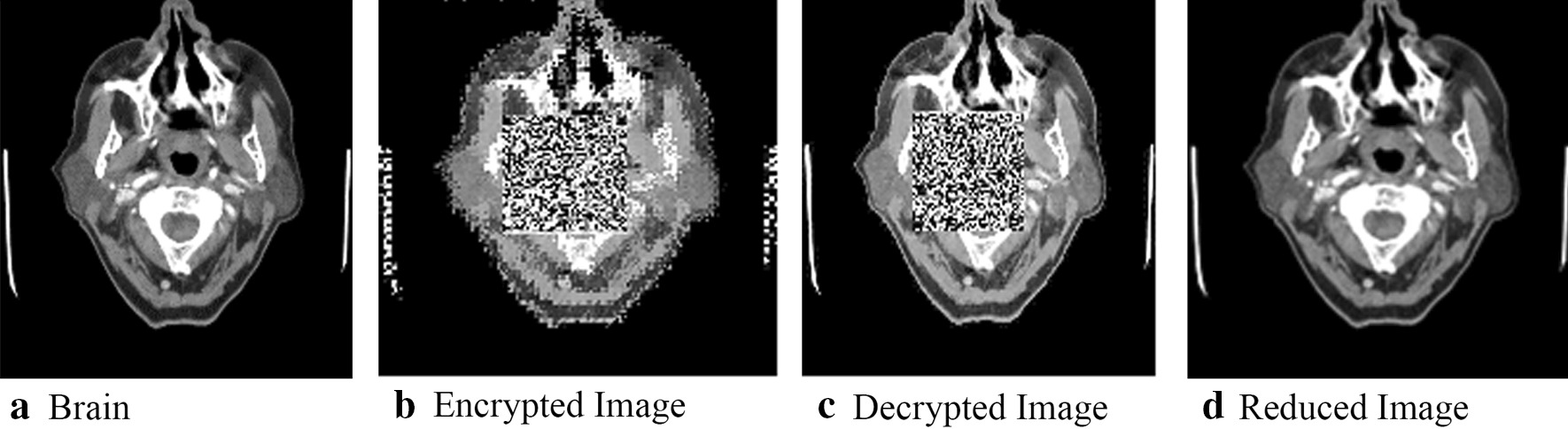
The encryption, decryption, and reduction of key brain image regions

**Fig. 6:**
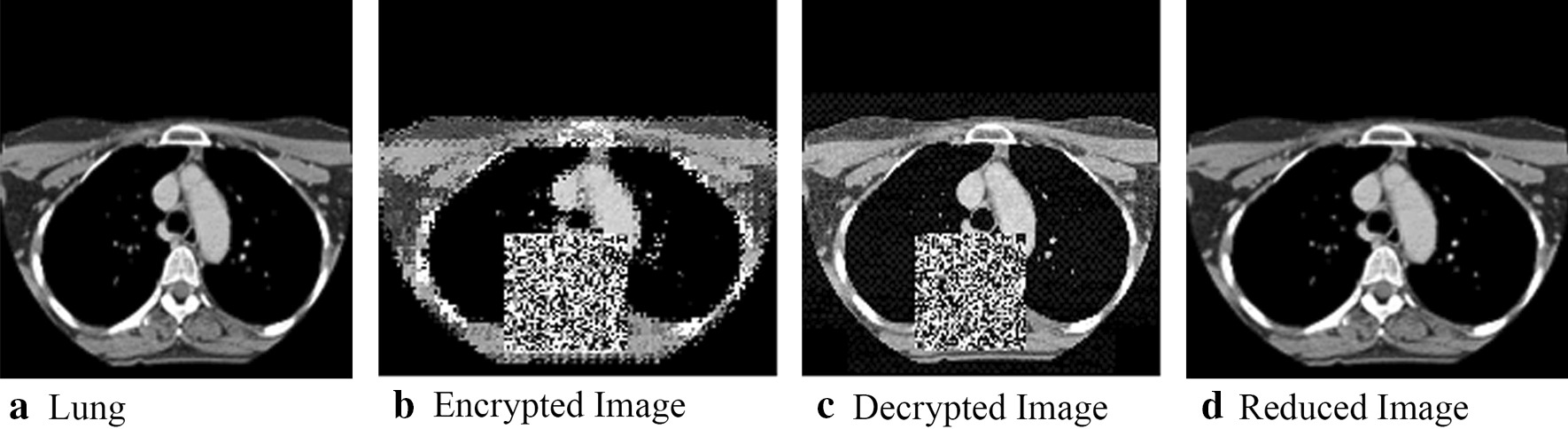
The encryption, decryption, and reduction of lung image

After we had ensured that the approach could detect and hide the key regions, we decide to conduct a more detailed analysis. We start with two of the three initial images; specifically, we display the stages of encryption and decryption for the initial brain and lung images, shown respectively in Figs. 5 and 6, with the original medical image shown in (5(a)/6(a)) and the encrypted image (5(b)/6(b)); mirroring what was shown in Fig. 4. 5(c)/6(c) shows scrambled decrypted image. Here, the image key area is covered with a QR code, and other high-texture areas embedded with secret information, resulting in an image visually different from the original. 5(d)/6(d) contains a reduced image following secret information extraction and image block reconstitution into a 160 × 160 block replacement for the QR code region.

While visual inspection is useful, we also collected measurements that indicate the degree of distortion achieved when encrypting images as well as the effectiveness of the reconstruction/decryption process. This evaluation was conducted on the three images shown in Fig. 4. Two measures were used: the PSNR and the Structural Similarity Index (SSIM). To calculate the two measures, we compared the original images to the encrypted image, for the decrypted imaged, and the final restored image [6]. The greater the PSNR value, the lesser the change in visual quality and degree of distortion following secret information embedding. An infinite PSNR would indicate that an image is recovered with no loss. Likewise, for SSIM, the greater the value, the greater the algorithm structural similarity. When SSIM is 1, this means the two images are identical in structure. We plot the values for PSNR and SSIM in Table 1. Based on the values of PSNR and SSIM, encrypted and decrypted images have only rough correspondence to the original images. This can largely attribute to the presence of the QR code, which hides the key region. However, both measures indicate the reconstructed images are perfect matches to the original images. Hence, our goals of hiding key information and achieving perfect reconstruction has been achieved.

**Table 1:** PSNR and SSIM of fully encrypted as well as restored medical images

	PSNR	SSIM
*Brain*		
Encryption	13.8033	0.4250
Decryption	15.8551	0.6632
Reduction	∞	1
*Lung*		
Encryption	12.9810	0.5404
Decryption	14.3018	0.7921
Reduction	∞	1
*Neck*		
Encryption	13.2778	0.4613
Decryption	14.7978	0.7487
Reduction	∞	1

### Key region for conservation

**Fig. 7:**
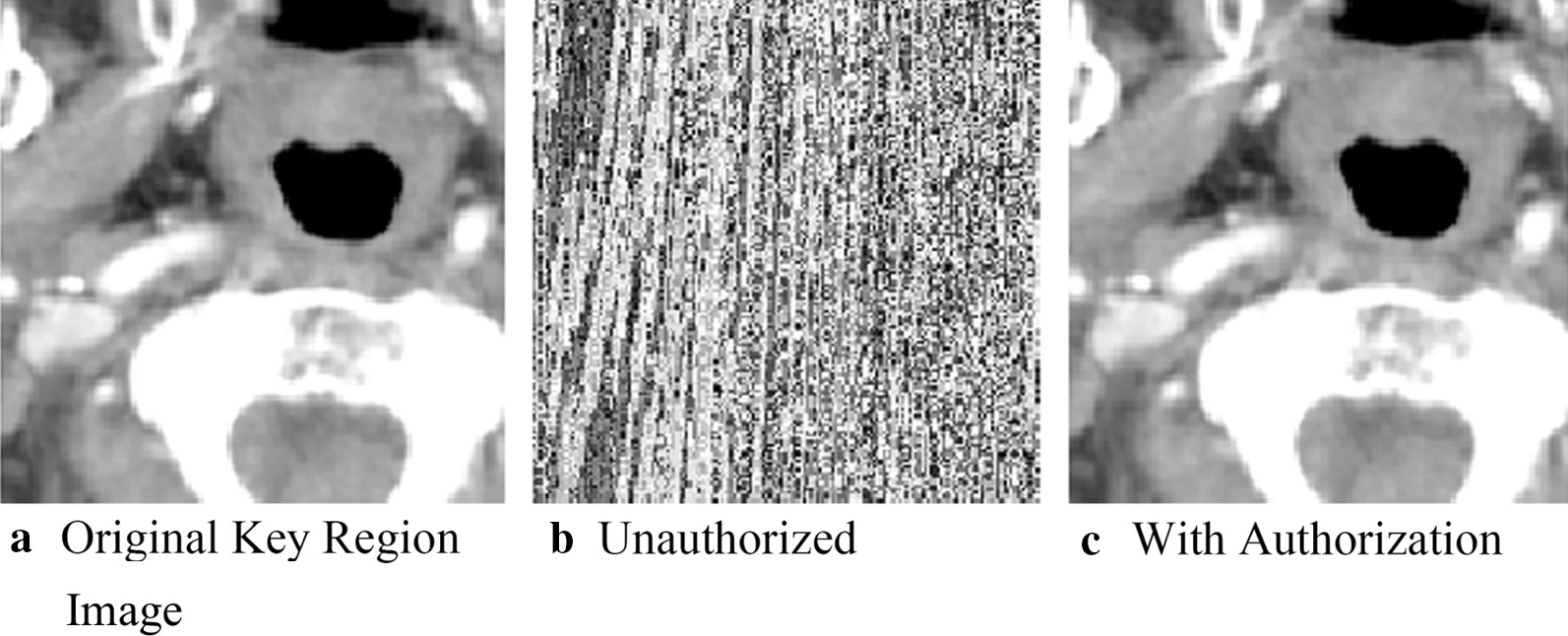
Key regions in brain image

**Fig. 8:**
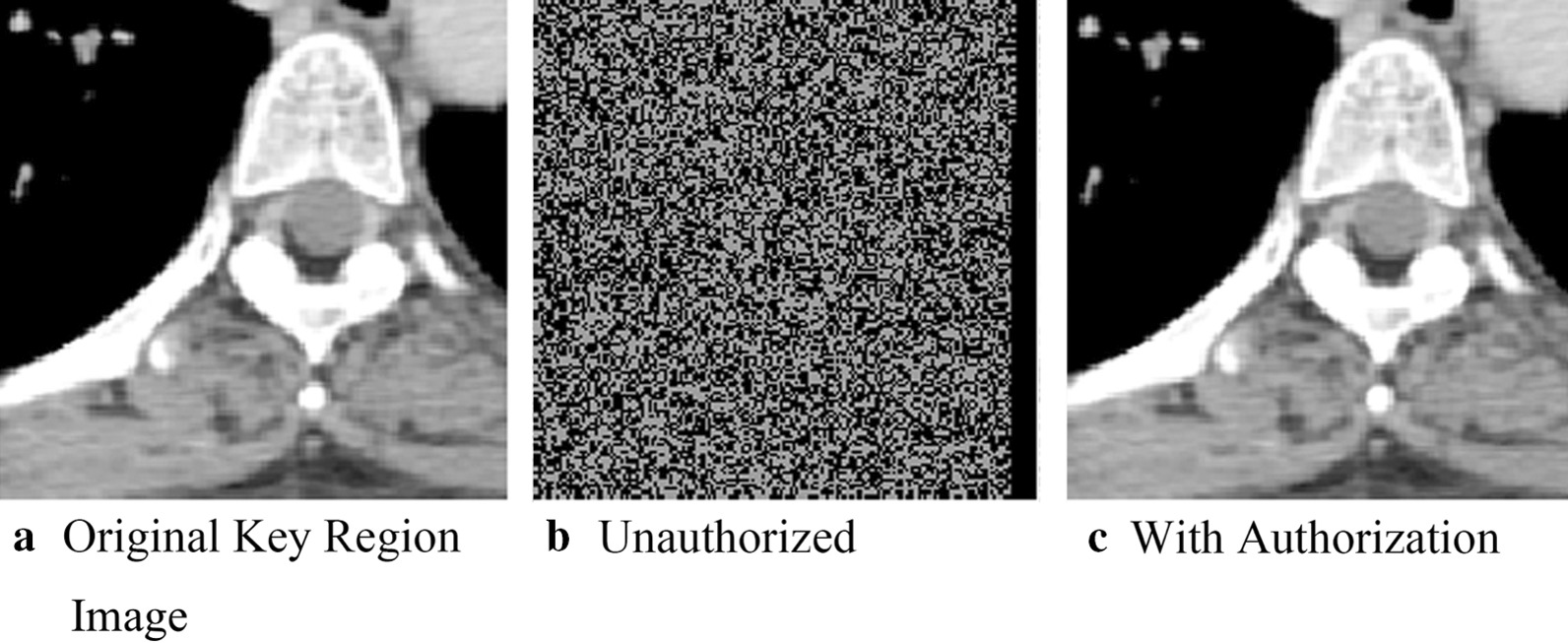
Key regions in lung images

While the above results indicate we can hide and recover key information, we want to ensure that unauthorized users could not successfully recover the key information. We visually represent this by showing the effects of authorized and unauthorized extraction for two images: one of the brain and one of the lung. These are shown respectively in Figs. 7 and 8. 7(a)/8(a) displays the key area of the original image for the brain and lung. 7(b)/8(b) shows what the key area looks like when extracted by an unauthorized user. It is visually obvious that no meaningful data have been obtained, indicating that we have successfully secured the key region. 7(c)/8(c) show the key area of the respective medical images when they are extracted/recovered by an authorized user. The key regions are visibility the same, which is unsurprisingly given the results shown in the "Embedded Image Extraction discussion."

In addition to visual inspection, we also calculated the PSNR and SSIM for images extracted by the unauthorized and authorized users against the original images of the key areas. We plot the values for PSNR and SSIM in Table 2. Notably, for the unauthorized extractions, the PSNR is less than ten, and SSIM less than 0.1. The structural similarity is completely different, blocking any unauthorized attempt to extract real information, and ensuring the real information of the image to be effectively protected; and the PSNR of key areas authorized for extraction is positive infinity. In addition, final SSIM values of one clearly indicate that images can be restored without loss after authorization.

**Table 2:** PSNR and SSIM of authorized and unauthorized extraction of medical image key areas

	PSNR	SSIM
*Brain*		
Unauthorized	7.8222	0.0093
Authorized	∞	1
*Lung*		
Unauthorized	7.2152	0.0182
Authorized	∞	1
*Neck*		
Unauthorized	7.0283	0.0060
Authorized	∞	1

### Comparison of using different threshold

**Fig. 9:**
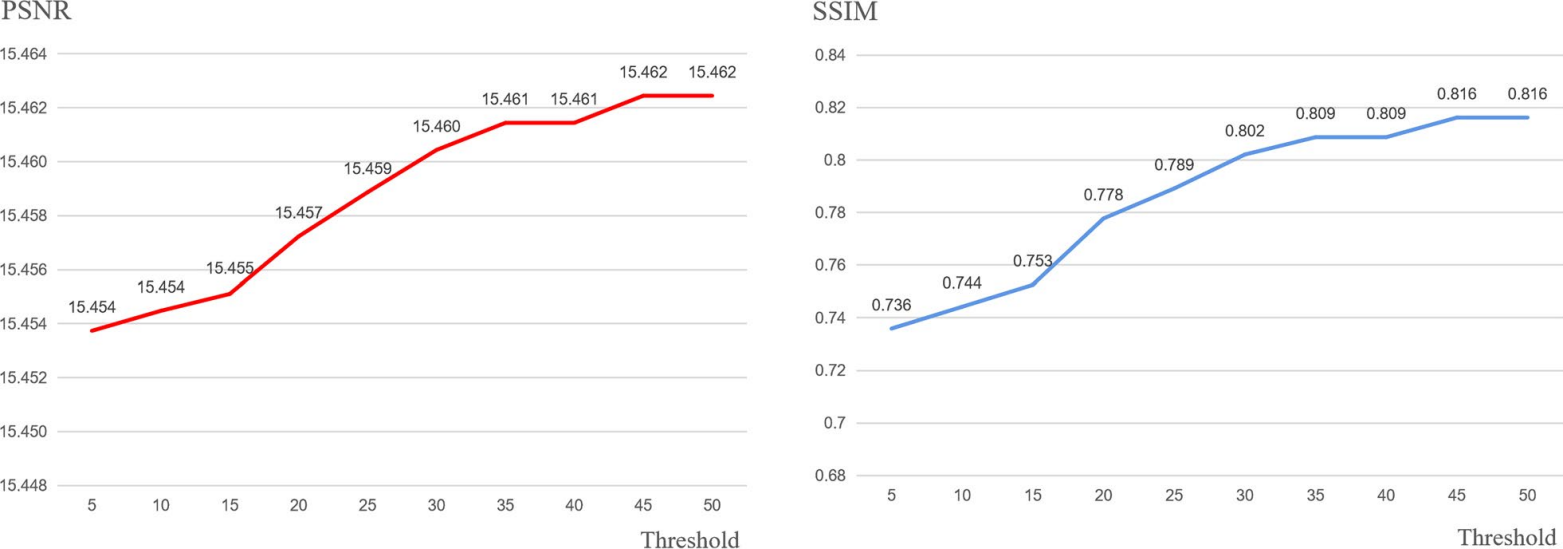
The PSNR and SSIM values of the encrypted brain image

**Fig. 10:**
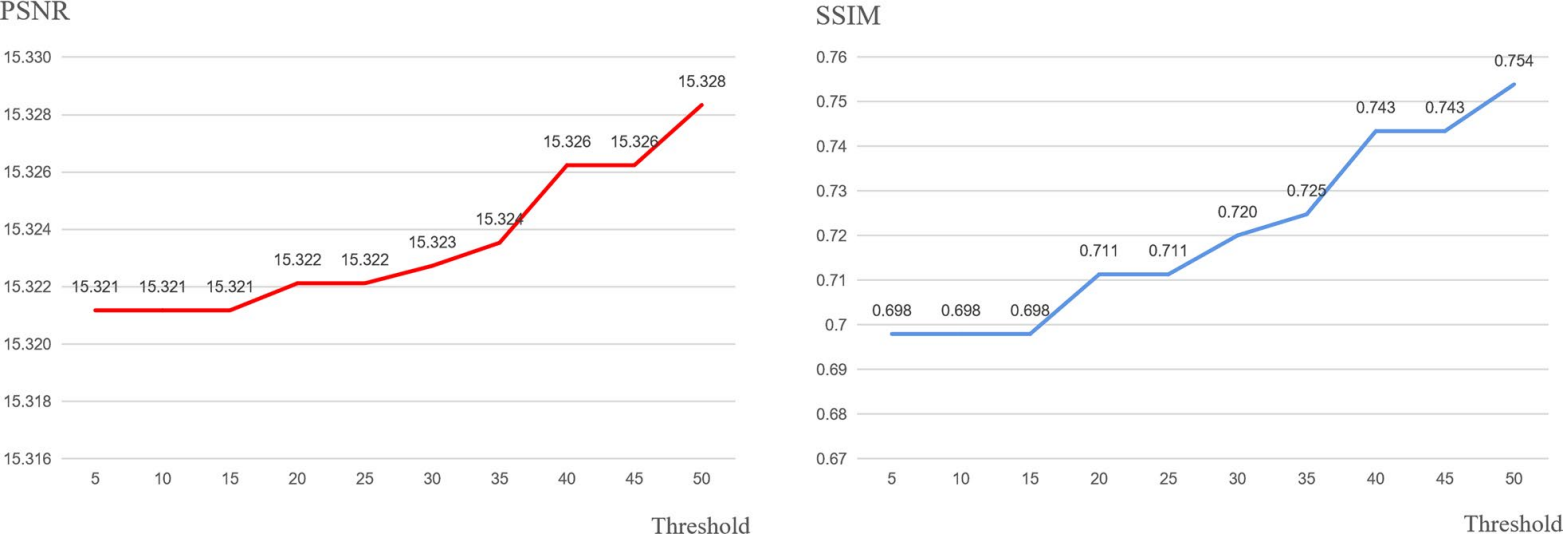
The PSNR and SSIM values of the encrypted lung image

**Fig. 11:**
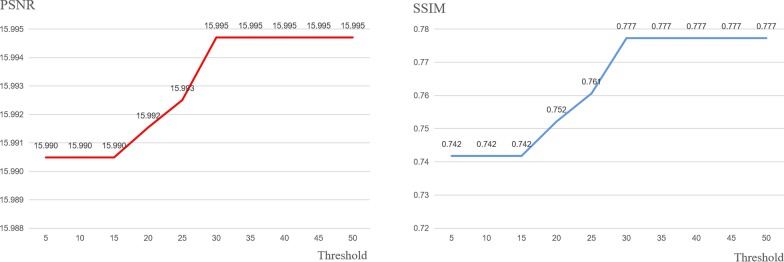
The PSNR and SSIM values of the encrypted neck image

Using different threshold T, Figs. 9, 10, and 11 show the PSNR and SSIM of brain, lung, and neck, respectively. In these figures, PSNR and SSIM plots increasing increments of 5 for thresholds between 5 and 50 on the x-axis. With the increase of threshold, the values of SSIM and PSNR increase continuously. It is because the selection of threshold determines the selection range of image blocks. The larger the threshold is, the less the number of selected embedded image blocks is, and the overall visual quality of the image is improved.

It can be seen from Figs. 9 and 11 that the PSNR (or SSIM) trend of brain and neck is gradually stable after the threshold reaches 45 and 30, respectively, unlike the instability shown in Fig. 10. The likely cause of this discrepancy is that the texture degree of each image is different, and thus, the range of MSE is also different. When the threshold value exceeds the maximum value of MSE, the image block with the maximum value will be automatically selected as the embedding area. In this case, the PSNR and SSIM will remain unchanged.

### Comparison of using different MSE

Table 3 shows the PSNR and SSIM of the encrypted image by selecting different MSE range in the key areas of one brain image, one lung image and one neck image compared to the original image. By adjusting the MSE range, we can know that the degradation effect can effectively protect the medical image.

**Table 3:** PSNR and SSIM of the encrypted image using different MSE range

	PSNR	SSIM
*Brain*		
[− 3,3]	15.8565	0.6758
[− 4,4]	15.8363	0.6611
[− 5,5]	15.8150	0.6456
[− 10,10]	15.8436	0.7571
[− 15,15]	15.8184	0.7514
[− 20,20]	15.7992	0.7514
*Lung*		
[− 3,3]	14.3019	0.7951
[− 4,4]	14.2845	0.7807
[− 5,5]	14.2662	0.7702
[− 10,10]	14.2635	0.8148
[− 15,15]	14.2327	0.8043
[− 20,20]	14.2118	0.7076
*Neck*		
[− 3,3]	14.7991	0.7569
[− 4,4]	14.8151	0.8267
[− 5,5]	14.8070	0.8222
[− 10,10]	14.7692	0.8022
[− 15,15]	14.7416	0.7960
[− 20,20]	14.7219	0.7936

### Comparison between image protection methods

Importantly, we have compared our methodology with two other routine methods of image protection. The first of these methods is an image encryption algorithm proposed by Goel and Chaudhari [31]. In this algorithm, the encrypted image content is completely invisible. This method enhances the security of the image, but it is difficult to retrieve the image. The second method we evaluated was that of Abdel-Nabi and Al-Haj [20] who proposed another information embedding algorithm, where the hidden information is embedded into the image. Table 4 shows comparisons of figure size, type, and PSNR. Of note, SSIM was not evaluated in the other two experiments and therefore not included in the comparison.

**Table 4:** Comparison of data protection methods

	Size	Type	PSNR
Proposed	512 × 512	Gray	15.8565
Goel and Chaudhari [31]	160 × 160	Gray	9.2895
Abdel-Nabi and Al-Haj [20]	512 × 512	Gray	58.8066

The PSNR of Goel and Chaudhari’s method is as low as 9.2895. The low PSNR value indicates that the encrypted image content is not visible to the naked eye and the image information is effectively encrypted. The PSNR of Abdel-Nabi and Al-Haj’s method is 58.8066. The higher PSNR means that the difference between the encrypted image and the original image is smaller, and image changes are difficult to detect by eye. That said, this method mainly aims at the protection of hidden information leaving the content security protection of the carrier image relatively vulnerable. In addition, this method discloses a lot of image information which is also not conducive to the safe transmission of medical images.

Compared with the above two methods, the PSNR of our algorithm is 15.8565. Of note, the purpose of our algorithm is to protect image information while ensuring the display of contour information. As such, in our method, we use both encryption and information hiding technologies. Although we obtain PSNRs somewhat higher than those obtained with Goel and Chaudhari’s encryption method, our use of QR codes avoids problems typically associated with their method including difficulties with encrypted image retrieval and vulnerability to attack. Furthermore, when we compare our method with that of Abdel-Nabi and Al-Haj’s, information hiding, we find our use of self-embedding and Arnold transformation similarly avoids the principle problem associated with this methodology: image leakage. Importantly, in contrast to Abdel-Nabi and Al-Haj’s method, our methodology removes the original background and protects the texture area containing information to improve the security of the image information and ensure only authorized users can obtain complete, intact image information.

## Conclusion

A novel process was presented for reversible data hiding for medical images. Our process allows for the automated detection of the key region of an image, the removal, encryption and embedding of the image within the larger medial images. Moreover, we have the ability to add to the transformed image, via QR codes, the transmission of basic patient and hospital information, along with encrypted information that allows for the efficient decoding of the image. The final version of the transformed image is the same size as the original image.

The primary drawback of the proposed approach is that the key to decode the encrypted information in the QR code must be known by authorized users. If lost, then recovery of the original image cannot be achieved from the transformed image. That said, this is an issue frequently encountered with any encryption scheme.

Future work will involve larger scale testing, both on the number of images as well as the metrics produced; the latter would include computational costs. We will examine the potential for using overlapping blocks to detect and encode a lesion area, instead of applying a one-time placement of non-overlapping blocks. This may confer a more robust detection and more tailored coverage and protection of key areas. Moreover, the current solution assumes a single region is sensitive; as such, we also plan to explore strategies for dealing with instances where multiple regions may be considered sensitive. Finally, we also plan to study the consequences of attacks aimed at compromising a transformed image along and to develop defenses to combat such events. Conceivably, malicious incursions could attempt to alter QR codes (encrypted and/or unprotected) as well as textured areas. As such, suitable defense strategies will need to include the ability to identify and recover from alterations resulting from such an attack.

## Data Availability

All images used for our experiment can be downloaded at: The Cancer Imaging Archive (TCIA): www.cancerimagingarchive.net. TCIA is an open-access database of medical images for cancer research. Patients are generally associated with a common disease (such as lung cancer), image morphology (MRI, CT, etc.), or research focus. Experimental data were obtained from LungCT-Diagnosis, LIDC-IDRI, and TCGA-GBM, etc.

## Abbreviations

BPP: Bit per pixel
C_v_: The coefficient of variation
CT: Computerized tomography
DE: Difference expansion
HS: Histogram shifting
MSE: Mean squared error
PEE: Prediction error expansion
PSNR: The peak signal to noise ratio
QR: Quick response
RDH: Reversible data hiding
SSIM: Structural Similarity Index
TCIA: The cancer imaging archive

## References

[1] Satoh H, Niki N, Eguchi K, Ohmatsu H, Kusumoto M, Kaneko M, Moriyama N. Teleradiology network system on cloud using the web medical image conference system with a new information security solution. In: SPIE medical imaging, vol. 8674. Lake Buena Vista (Orlando Area): SPIE; 2013.

[2] Avudaiappan T, Balasubramanian R, Pandiyan SS, Saravanan M, Lakshmanaprabu SK, Shankar K (2018). Medical image security using dual encryption with oppositional based optimization algorithm. J Med Syst.

[3] Wang C, Wang X, Xia Z, Zhang C (2019). Ternary radial harmonic Fourier moments based robust stereo image zero-watermarking algorithm. Inf Sci.

[4] Method and apparatus for embedding authentication information within digital data. https://patents.google.com/patent/US5646997A/en. Accessed 24 April 2019.

[5] Ma B, Wang X, Li Q, Li B, Li J, Wang C, Shi Y (2019). Adaptive error prediction method based on multiple linear regression for reversible data hiding. J Real-Time Image Proc.

[6] Ma B, Li B, Wang X-Y, Wang C-P, Li J, Shi Y-Q (2019). A code division multiplexing and block classification-based real-time reversible data-hiding algorithm for medical images. J Real-Time Image Process.

[7] Ma B, Li B, Wang X-Y, Wang C, Li J, Shi YQ (2019). Code division multiplexing and machine learning based reversible data hiding scheme for medical image. Secur Commun Netw.

[8] Shi Y, Li X, Zhang X, Wu H, Ma B (2016). Reversible data hiding: advances in the past two decades. IEEE Access.

[9] Ma B, Shi YQ (2016). A reversible data hiding scheme based on code division multiplexing. IEEE Trans Inf Forensics Secur.

[10] Gurusamy R, Subramaniam V (2017). A machine learning approach for MRI brain tumor classification. Comput Mater Continua.

[11] Celik MU, Sharma G, Tekalp AM, Saber E (2005). Lossless generalized-lSB data embedding. IEEE Trans Image Process.

[12] Jun T (2003). Reversible data embedding using a difference expansion. IEEE Trans Circuits Syst Video Technol.

[13] Zhicheng N, Yun-Qing S, Ansari N, Wei S (2006). Reversible data hiding. IEEE Trans Circuits Syst Video Technol.

[14] Kumar CV, Natarajan V, Bhogadi D. High capacity reversible data hiding based on histogram shifting for medical images. In: 2013 international conference on communication and signal processing: 3–5 April 2013; Tamilnadu; 2013, p. 730–3.

[15] Yang Y, Zhang W, Yu N. Improving visual quality of reversible data hiding in medical image with texture area contrast enhancement. In: 2015 international conference on intelligent information hiding and multimedia signal processing (IIH-MSP): 23–25 Sept 2015; Adelaide; 2015. p. 81–4.

[16] Wu M, Zhao J, Chen B, Zhang Y, Yu Y, Cheng J. Reversible data hiding based on medical image systems by means of histogram strategy. In: 2018 3rd international conference on information systems engineering (ICISE): 4–6 May 2018; Shanghai; 2018. p. 6–9.

[17] Huang L-C, Tseng L-Y, Hwang M-S (2013). A reversible data hiding method by histogram shifting in high quality medical images. J Syst Softw.

[18] Norcen R, Podesser M, Pommer A, Schmidt HP, Uhl A (2003). Confidential storage and transmission of medical image data. Comput Biol Med.

[19] Brahimi Z, Bessalah H, Tarabet A, Kholladi M-K. Selective encryption techniques of JPEG2000 codestream for medical images transmission, vol. 7; 2008.

[20] Abdel-Nabi H, Al-Haj A. Medical imaging security using partial encryption and histogram shifting watermarking. In: 2017 8th international conference on information technology (ICIT): 17–18 May 2017; Amman; 2017. p. 802–7.

[21] Tiwari S. An introduction to QR code technology. In: 2016 international conference on information technology (ICIT): 22–24 Dec 2016; Bhubaneswar; 2016. p. 39–44.

[22] Li J, Zhang Z, Li S, Benton R, Huang Y, Kasukurthi MV, Li D, Lin J, Borchert GM, Tan S et al. Reversible data hiding based key region protection method in medical images. In: 2019 IEEE international conference on bioinformatics and biomedicine (BIBM-19): 2019; San Diego; 2019. p. 1526–30.

[23] Schnev V, Kim HJ, Nam J, Suresh S, Shi YQ (2010). Reversible watermarking algorithm using sorting and prediction. IEEE Trans Circuits Syst Video Technol.

[24] Puech W. Image encryption and compression for medical image security. In: International workshops on image processing theory, tools and applications: 23–26 Nov 2008: Sousse; 2008. p. 1–2.

[25] Wang XY, Ma B, Li J, Shi Y-Q (2018). Adaptive image reversible data hiding error prediction algorithm based on multiple linear regression. Yingyong Kexue Xuebao/J Appl Sci.

[26] Veena VK, Lal GJ, Prabhu SV, Kumar SS, Soman KP. A robust watermarking method based on compressed sensing and Arnold scrambling. In: 2012 international conference on machine vision and image processing (MVIP): 14–15 Dec 2012. p. 105–8.

[27] Feng M-A, Feng B, Shen C (2008). Adaptive image watermarking algorithm based on block DCT transform and Arnold shuffling. J Comput Appl.

[28] Saha BJ, Pradhan C, Kabi KK, Bisoi AK. Robust watermarking technique using Arnold's transformation and RSA in discrete wavelets. In: 2014 international conference on information systems and computer networks (ISCON): 1–2 Mar 2014; 2014. p. 83–7.

[29] Arnold VI, Avez A (1970). Ergodic problems of classical mechanics.

[30] Yue L, Ju Y, Mingjun L. Recognition of QR code with mobile phones. In: 2008 Chinese control and decision conference: 2–4 July 2008; Yantai; 2008. p. 203–6.

[31] Goel A, Chaudhari K. FPGA implementation of a novel technique for selective image encryption. In: 2016 2nd international conference on frontiers of signal processing (ICFSP): 15–17 Oct 2016 2016; 2016. p. 15–9.

